# Draft genome sequences data of *Mammaliicoccus lentus* isolated from horse farm soil

**DOI:** 10.1016/j.dib.2023.109752

**Published:** 2023-11-04

**Authors:** Pavel Alexyuk, Madina Alexyuk, Yergali Moldakhanov, Vladimir Berezin, Andrey Bogoyavlenskiy

**Affiliations:** Research and Production Center for Microbiology and Virology, Bogenbay batyr. Str., 105, Almaty 050010, Kazakhstan

**Keywords:** Staphylococcaceae, Coagulase-negative bacteria, Sequencing, Genome annotation

## Abstract

*Mammallicoccus lentus* is a member of the commensal microflora of the *Staphylococcaceae* family, which colonizes the skin of several species of farm animals, including poultry and dairy animals (Huber et al., 2011; Zhang et al., 2009). The study of the members of the *Staphylococcaceae* family, such as the *Mammaliicoccus* genus, isolated from various sources is of great importance for agriculture and public health as contributes to the accumulation of knowledge and understanding of the mechanisms of antibiotic resistance gene transmission among bacterial pathogens. This thesis is supported by recent studies showing that some members of the *Mammallicoccus* genus serve as a reservoir of virulence and antibiotic resistance genes and may also be a source of horizontal gene transfer (Saraiva et al., 2021). Here, we present a draft genome sequence of *Mammallicoccus lentus* strain PVZ.22 from a horse farm soil sample. The sequencing was performed on the Illumina MiSeq platform. The genome was assembled using the Geneious software package. The genome contains 2,802,282 bp with a total of 2805 genes, 8 perfect and 12 strict AMR genes and 58 tRNAs genes.

Specifications Table

**Table:** 

Subject	Microbiology
Specific subject area	Microbial genomics
Data format	Raw reads and analysed genome of *Mammaliicoccus lentus* PVZ.22
Type of data	Table, figure
Data collection	DNA extraction was performed using the PureLink Genomic DNA Mini Kit (Invitrogen, USA). DNA was quantified and checked for by Tecan's NanoQuant Plate. DNA library was prepared using Nextera XT DNA sample preparation kit Sequencing was performed on the Illumina MiSeq sequencing platform.
Data source location	Research and Production Center for Microbiology and Virology • City/Region: Almaty • Country: Kazakhstan • Latitude and longitude: 43°15′14.2"N 76°57′11.1"E
Data accessibility	Direct URL to BioSample: https://www.ncbi.nlm.nih.gov/biosample/SAMN32925311 Direct URL to BioProject: https://www.ncbi.nlm.nih.gov/bioproject/?term=PRJNA928237 Direct URL to Whole Genome Data: https://www.ncbi.nlm.nih.gov/nuccore/NZ_CP116807.1 Direct URL to Raw Sequencing Data: https://data.mendeley.com/datasets/pv29k73dc4/2

## 1 Value of the Data

• The complete genome sequence of *Mammaliicoccus lentus* isolated from horse farm soil provides information that may be helpful for understanding the epidemiology of bacterial infections in domestic animals.
• The analyses of antibiotic resistance genes can be used to predict the probability of the strain being a multidrug resistance pathogen.
• The data may assist researchers in study of antimicrobial resistance genes.
• The data can be used in study of bacterial pathogenicity evolution.
• The data can be used by researchers for genomics and other evolutionary studies.

## 2 Data Description

**Fig. 1:**
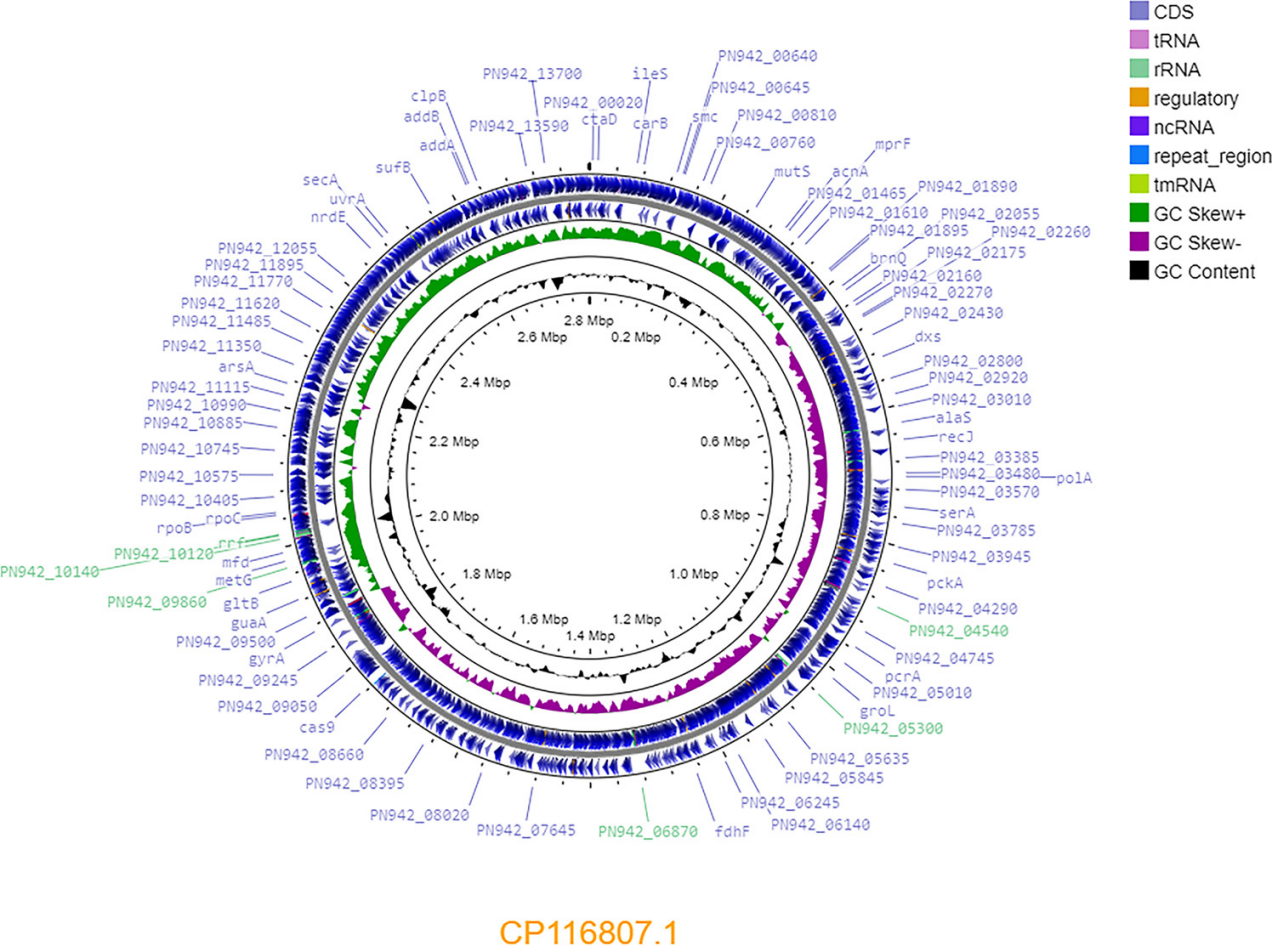
The genome map of *Mammaliicoccus lentus* strain PVZ.22 built using CGView server (https://proksee.ca/assessed on 18th August 2023). The blue arrows represent CDSs; green peaks represent GC-skew+; purple represents GC-skew-; and black peaks represent G+C content.

The *Mammaliicoccus* genus was separated from *Staphylococcus* into an independent genus in 2020. It includes 5 species (*Mammaliicoccus sciuri, Mammaliicoccus lentus, Mammaliicoccus vitulinus, Mammaliicoccus fleurettii* and *Mammaliicoccus stepanovicii*), which are most often commensal bacteria associated with animals [[1], [2], [3], [4], [5]]. Here we present data on the *Mammaliicoccus lentus* strain PVZ.22 genome. Table 1 and Fig. 1 show brief characteristics of the *Mammaliicoccus* genome.

**Table 1:** Genome characteristics of the *Mammaliicoccus lentus* PVZ.22.

Features	PVZ.22
Genome size (bp)	2,802,282
GC% content	31.90
Number of contigs	444
N50 length (bp)	69,073
Genes (total)	2,805
CDSs (total)	2,724
Genes (RNA)	81
tRNAs	58
CRISPR Arrays	1
Resistance genes	present

Taxonomic affiliation of the strain was predicted by whole genome comparison using the genome-to-genome distance calculator (GGDC) and OrthoANI values. The most closely related microorganism found in the GenBank database was Mammaliicoccus lentus strain H29 (Table 2).

**Table 2:** Results of whole genome comparison of *Mammallicoccus lentus* strain PVZ. 22.

Strain	Accession	Size (bp)	GC%	OrthoANI value (%)	GGDC distance
*Mammaliicoccus lentus* strain H29	CP059679.1	2,802,282	31.90	99.58	0.0041
*Mammaliicoccus lentus* strain Colony430	CP075504.1	2,802,382	32.07	99.48	0.0826
*Mammaliicoccus lentus* strain 7074	CP118776.1	2,590,488	31.95	99.18	0.0848
*Mammaliicoccus sciuri* strain GDK8D6P	CP065792.1	2,726,042	32.52	80.76	0.416
*Mammaliicoccus vitulinus* strain DSM 15615	CP118974.1	2,643,161	32.65	80.89	0.4098

The genome was screened for antimicrobial resistance genes against the Comprehensive Antibiotic Resistance Database (CARD; card.mcmaster.ca). The predicted resistance genes in the genome are presented in Table 3.

**Table 3:** Predicted antimicrobial resistance genes of *Mammaliicoccus lentus* PVZ.22.

RGI Criteria	Detection Criteria	AMR Gene Family	Drug Class	Resistance Mechanism	% Identity of Matching Region	% Length of Reference Sequence
Strict	vanY gene in vanM cluster	vanY, glycopeptide resistance gene cluster	glycopeptide antibiotic	antibiotic target alteration	33.51	101.72
Strict	vanT gene in vanG cluster	glycopeptide resistance gene cluster, vanT	glycopeptide antibiotic	antibiotic target alteration	32.34	53.65
Strict	sepA	small multidrug resistance (SMR) antibiotic efflux pump	disinfecting agents and antiseptics	antibiotic efflux	54.86	98.73
Strict	sdrM	major facilitator superfamily (MFS) antibiotic efflux pump	fluoroquinolone antibiotic, disinfecting agents and antiseptics	antibiotic efflux	47.26	99.11
Strict	salB	sal-type ABC-F protein	lincosamide antibiotic, streptogramin antibiotic, streptogramin A antibiotic, pleuromutilin antibiotic	antibiotic target protection	99.08	100.00
Strict	mphC	macrolide phosphotransferase (MPH)	macrolide antibiotic	antibiotic inactivation	90.97	100.00
Strict	mecA	methicillin resistant PBP2	penam	antibiotic target replacement	99.85	100.00
Strict	FosBx1	fosfomycin thiol transferase	phosphonic acid antibiotic	antibiotic inactivation	55.8	109.42
Strict	ErmB	Erm 23S ribosomal RNA methyltransferase	macrolide antibiotic, lincosamide antibiotic, streptogramin antibiotic, streptogramin A antibiotic, streptogramin B antibiotic	antibiotic target alteration	98.78	98.79
Strict	ErmB	Erm 23S ribosomal RNA methyltransferase	macrolide antibiotic, lincosamide antibiotic, streptogramin antibiotic, streptogramin A antibiotic, streptogramin B antibiotic	antibiotic target alteration	97.96	98.79
Strict	aad(6)	ANT(6)	aminoglycoside antibiotic	antibiotic inactivation	100.0	109.42
Strict	AAC(6′)-Ie-APH(2′')-Ia bifunctional protein	aminoglycoside bifunctional resistance protein	aminoglycoside antibiotic	antibiotic inactivation	99.79	102.51
Perfect	mecR1	methicillin resistant PBP2	penam	antibiotic target replacement	100.0	100.00
Perfect	mecI	methicillin resistant PBP2	penam	antibiotic target replacement	100.0	100.00
Perfect	FosD	fosfomycin thiol transferase	phosphonic acid antibiotic	antibiotic inactivation	100.0	100.00
Perfect	FosD	fosfomycin thiol transferase	phosphonic acid antibiotic	antibiotic inactivation	100.0	100.00
Perfect	FosD	fosfomycin thiol transferase	phosphonic acid antibiotic	antibiotic inactivation	100.0	100.00
Perfect	fexA	major facilitator superfamily (MFS) antibiotic efflux pump	phenicol antibiotic	antibiotic efflux	100.0	100.00
Perfect	dfrG	trimethoprim resistant dihydrofolate reductase dfr	diaminopyrimidine antibiotic	antibiotic target replacement	100.0	100.00
Perfect	cfrA	Cfr 23S ribosomal RNA methyltransferase	lincosamide antibiotic, streptogramin antibiotic, streptogramin A antibiotic, oxazolidinone antibiotic, phenicol antibiotic, pleuromutilin antibiotic	antibiotic target alteration	100.0	100.00

## 3 Experimental Design, Materials and Methods

*Mammaliicoccus lentus* strain PVZ.22 was isolated from horse farm soil in Kazakhstan (43.184873 N 76.523994 E). The pure isolate was cultured in commercially prepared tryptic soy agar (Conda, Spain) with 5 % sheep red blood cells. The initial species identification was carried out by visually (non-hemolytic, white opaque colonies) and light microscopy (Gram positive cocci clustered in grape-like aggregates) [6,7].

For DNA extraction, culture was grown in nutrient broth (Conda, Spain) for 24 h at 37 °C. The DNA was extracted using PureLink Genomic DNA Mini Kit (Invitrogen, USA) according to the manufacturer's protocol. The quality of the resulting DNA was determined using the Tecan's NanoQuant Plate (Tecan, Männedorf, Switzerland) at the optical wavelengths of 260 and 280 nm. The DNA library was prepared using the Nextera XT DNA sample preparation kit (Illumina, Cambridge, UK), and quantified using the Qubit 3.0 and Qubit dsDNA High Sensitivity Kit (Invitrogen, USA). The library was then sequenced on the Illumina MiSeq platform with 2  ×  300 bp reads. The raw Illumina reads were trimmed and assembled de novo with with SPAdes 3.12.0 (an installable plugin in Geneious Prime) [8], [9], [10]. A complete genome of *Mammaliicoccus lentus* strain H29 chromosome (CP059679) was used as reference genome. The annotation was added by the NCBI Prokaryotic Genome Annotation Pipeline (PGAP) [11]. Information about PGAP can be found here: https://www.ncbi.nlm.nih.gov/genome/annotation_prok/. The Resistance Gene Identifier (RGI v5.2.0) was used for screening of antimicrobial resistance genes [12]. The OrthoANIu tool [13] was used to compare prokaryotic genome sequence when classifying and identifying bacteria (https://www.ezbiocloud.net/tools/ani

(assessed 17.08.2023). The identification of CRISPR arrays and their associated Cas proteins was performed using the CRISPRCasFinder 4.2.20 software [14].

The raw reads are available online at the Mendeley repository [15]. The complete chromosomal sequence was deposited under the accession number - NZ_CP116807.

## Limitations

‘Not applicable’.

## Ethics Statements

Work did not include animal experiments or data collected from social media platforms or human subjects.

## CRediT authorship contribution statement

**Pavel Alexyuk:** Conceptualization, Software, Validation, Writing – original draft, Writing – review & editing. **Madina Alexyuk:** Data curation, Writing – review & editing. **Yergali Moldakhanov:** Investigation. **Vladimir Berezin:** Supervision. **Andrey Bogoyavlenskiy:** Methodology, Software, Writing – original draft.

## Data Availability

Raw sequence data (Original data).
